# Exercise Counteracts Aging-Related Memory Impairment: A Potential Role for the Astrocytic Metabolic Shuttle

**DOI:** 10.3389/fnagi.2016.00057

**Published:** 2016-03-22

**Authors:** Sheng-Feng Tsai, Pei-Chun Chen, Marcus J. Calkins, Shih-Ying Wu, Yu-Min Kuo

**Affiliations:** ^1^Institute of Basic Medical Sciences, College of Medicine, National Cheng Kung UniversityTainan, Taiwan; ^2^Department of Physiology, College of Medicine, National Cheng Kung UniversityTainan, Taiwan; ^3^Institute of Clinical Medicine, College of Medicine, National Cheng Kung UniversityTainan, Taiwan; ^4^Department of Cell Biology and Anatomy, College of Medicine, National Cheng Kung UniversityTainan, Taiwan

**Keywords:** lactate, monocarboxylate transporter, glutamate transporter, lactate dehydrogenase, hydroxycarboxylic acid receptor

## Abstract

Age-related cognitive impairment has become one of the most common health threats in many countries. The biological substrate of cognition is the interconnection of neurons to form complex information processing networks. Experience-based alterations in the activities of these information processing networks lead to neuroadaptation, which is physically represented at the cellular level as synaptic plasticity. Although synaptic plasticity is known to be affected by aging, the underlying molecular mechanisms are not well described. Astrocytes, a glial cell type that is infrequently investigated in cognitive science, have emerged as energy suppliers which are necessary for meeting the abundant energy demand resulting from glutamatergic synaptic activity. Moreover, the concerted action of an astrocyte-neuron metabolic shuttle is essential for cognitive function; whereas, energetic incoordination between astrocytes and neurons may contribute to cognitive impairment. Whether altered function of the astrocyte-neuron metabolic shuttle links aging to reduced synaptic plasticity is unexplored. However, accumulated evidence documents significant beneficial effects of long-term, regular exercise on cognition and synaptic plasticity. Furthermore, exercise increases the effectiveness of astrocyte-neuron metabolic shuttle by upregulation of astrocytic lactate transporter levels. This review summarizes previous findings related to the neuronal activity-dependent astrocyte-neuron metabolic shuttle. Moreover, we discuss how aging and exercise may shape the astrocyte-neuron metabolic shuttle in cognition-associated brain areas.

## Introduction

Aging is an irreversible and inescapable process. Improving medical care and falling fertility rates are resulting in population aging worldwide. According to a report by the World Health Organization, the number of people aged 65 or older is expected to grow from an estimated 524 million in 2010 to nearly 1.5 billion in 2050, representing 16 percent of world’s population. Consequently, aging-related dementia has become one of the greatest health threats in many countries, with a worldwide cost in 2010 exceeding US$ 600 billion, according to Alzheimer’s disease (AD) International. Memory loss is one of the most daunting problems among the symptoms of dementia. Learning and memory are the processes in which external environmental information are encoded, stored and retrieved. It is widely assumed that changing the strength of connections between neurons is essential for encoding and storing memory traces in the central nervous system (CNS) (Martin et al., 2000). These alterations in properties of neuronal transmission are termed synaptic plasticity. Activity-dependent synaptic plasticity is induced at appropriate synapses during learning and memory processes, and is necessary for information storage. Indeed, synaptic plasticity is known to be negatively affected by aging, but the mechanisms underlying the association between aging-related memory deficits and synaptic plasticity are relatively undescribed (Bishop et al., 2010).

Energy demand is dramatically increased during synaptic transmission and activity-dependent synaptic plasticity. Excitatory neurotransmitters are released from presynaptic neurons into the synaptic cleft and stimulate postsynaptic neurons by acting on postsynaptic receptors. Abundant energy is needed in order to execute receptor-mediated signal transduction, receptor trafficking, neurotransmitter synthesis, vesicle filling, release, uptake and recycling (Khatri and Man, 2013). Astrocytes, a glial cell type that is infrequently investigated in cognitive science, have emerged as energy suppliers which are necessary for meeting the ample energy demand resulting from synaptic activity (Belanger et al., 2011). Compared to neurons, astrocytes exhibit higher rates of glycolysis, but lower rates of oxidative phosphorylation (Itoh et al., 2003; Herrero-Mendez et al., 2009). Furthermore, a large portion of glucose, the dominant and essential energy source in the adult brain, which enters the glycolytic pathway in astrocytes is released as lactate into the extracellular space (Pellerin and Magistretti, 1994, 1997; Bouzier-Sore et al., 2006). The astrocytic glucose-derived lactate is known to fill the increased energy needs during synaptic transmission. The transportation of lactate between astrocytes and neurons is the so-called astrocyte-neuron lactate shuttle (ANLS). Correct functioning of the ANLS is essential for the manifestation of cognitive function; while poor energetic synchronization between astrocytes and neurons may contribute to cognitive impairment. However, the effect of aging on ANLS is largely undescribed.

Long-term, regular physical exercise is widely known to produce effects which combat various pathological conditions, especially cardiovascular disease and metabolic abnormalities. In past decades, numerous cohort studies have also documented that exercise enhances cognitive function, and delays the onset of aging-related dementia in humans (Fernández-Tomé et al., 2004; Frizzo et al., 2004). Similarly, animal studies have provided evidence for the protective role of exercise in memory deficits during aging (Diamond, 2005; Tramontina et al., 2006; Zeng et al., 2007). Moreover, exercise is known to affect functions related to the ANLS, such as stimulation of lactate release and oxidation in the brain (Overgaard et al., 2012) and elevation of lactate and glucose transporter expression (Takimoto and Hamada, 2014). These results suggest that sustained exercise may be a potential strategy to prevent dementia and that the ANLS may be involved in producing exercise-improved memory function during aging. To explore this concept, this review summarizes the known mechanisms which comprise the astrocyte-neuron metabolic shuttle during neuronal activity and how aging and exercise may help to shape the astrocyte-neuron metabolic shuttle in brain areas associated with learning and memory. We begin with a description of the ANLS and its role in memory formation. Then, we discuss aging-induced alterations of brain metabolism that may underlie memory deficits, with a focus on the potential role of the ANLS. To begin to understand whether regular exercise benefits aging-related memory impairments by improving the metabolic functions, we discuss how regular exercise regulates the ANLS functions. We hope that the growing understanding of the molecular basis of the ANLS and the interactions between exercise and aging in the astrocyte-neuron metabolic shuttle will allow us to develop strategies for treating and preventing aging-related memory decline.

## Astrocyte-Neuron Lactate Shuttle

The brain is the most energy-demanding organ in human body. It consumes 20% of the oxygen, 25% of glucose and approximately 20% of the total ATP supply, yet comprises only 2% of the total body mass (Attwell and Laughlin, 2001). Although glial cells outnumber neurons by about tenfold, almost 85% of the brain’s energy is consumed by neurons (Khatri and Man, 2013). Neural activity is comprised of two critical events, propagation of action potentials and synaptic transmission. For many years, researchers have debated which functions represent primary energy consuming processes in neurons (Alle et al., 2009; Howarth et al., 2012). Recent studies have demonstrated that the generation and spread of action potentials are relatively efficient (Alle et al., 2009; Howarth et al., 2012), and a large amount of energy is used to support synaptic transmission. Neurotransmitter synthesis, vesicle filling, release, uptake, and recycling, as well as the postsynaptic receptor trafficking and further signal transduction are all ATP consuming processes (Jolivet et al., 2009; Howarth et al., 2012). It has been estimated that, in the cerebral cortex about 50% of energy is used in the regulation of postsynaptic glutamate receptors, 21% in action potentials, 20% in maintaining resting potentials, 5% in presynaptic transmitter release, and 4% in transmitter recycling (Attwell and Laughlin, 2001). In the cerebellum, 54% of energy is used in the maintenance of resting potentials, 22% in regulating postsynaptic receptors, and 17% in action potentials (Attwell and Laughlin, 2001).

To meet the energy demands for neuronal activation, energy substrates must be delivered with precise spatiotemporal coordination. In other words, the demand and supply of energy need to be tightly coupled for the proper execution of brain function (Petzold and Murthy, 2011; Zlokovic, 2011). It is conventionally thought that the increases of neuronal activity are accompanied by an increase of local blood flow and an elevation of neuronal glucose consumption, the dominant and essential energy source in the adult brain. However, a series of positron emission tomography (PET) studies suggested that this may be an oversimplification of the tissue metabolic response. In the mid-1980s, Fox and colleagues found that the neuronal firing-dependent increases of brain blood flow and glucose consumption exceeded the elevation in oxygen consumption in awake, adult humans (Fox and Raichle, 1986; Fox et al., 1988). Their findings implied that non-oxidative glycolysis is also involved in producing energy after neuronal activation. This conjecture was later supported by a study using ^1^H-nuclear magnetic resonance (NMR) spectroscopy in healthy adults which indicated that level of lactate, an end product of non-oxidative glycolysis, increases with corresponding elevations in brain activity (Figley and Stroman, 2011). Lactate was formerly believed to be harmful to the brain; thus, its clearance by cerebrospinal fluid circulation or glial cells was thought to be necessary following neuronal activation. However, this concept was challenged with identification of the lactate oxidation complex in neurons, which allows lactate to enter into mitochondria and promotes oxidation (Hashimoto et al., 2008). It is now widely accepted that the lactate generated by glycolysis can be used as an energy source by neurons during neurotransmission.

Among the glial cells which support neuronal functions, astrocytes have emerged as active players in brain energy delivery, production, utilization, and storage (Belanger et al., 2011). Astrocytes cooperate with neurons in energy metabolism by preferentially utilizing complementary metabolic substrates. To meet the high energy requirements imposed by cellular function, neurons efficiently generate ATP primarily through oxidative phosphorylation rather than relying on glycolysis (Itoh et al., 2003). One major control point for glycolysis is activity of phosphofructokinase-1 (PFK-1), which is strongly stimulated by the metabolic product fructose-2,6-bisphosphate. It has been reported that the enzyme 6-phosphofructose-2-kinase/fructose-2,6- bisphosphatase-3 (Pfkfb3) which governs the generation of fructose-2,6-bisphosphate is virtually absent in neurons due to high turnover and efficient proteasomal degradation (Herrero-Mendez et al., 2009). In contrast, astrocytes abundantly express Pfkfb3 and exhibit relatively high levels of glycolysis due to the allosteric activation of PFK-1 by fructose-2,6-bisphosphate (Itoh et al., 2003; Herrero-Mendez et al., 2009). While a portion of the pyruvate produced by astrocytic glycolysis is converted to lactate and excreted, it is worth mentioning that the citric acid cycle substantially contributes to ATP generation in astrocytes. Recent NMR spectroscopy studies have reported that astrocytes account for more than 20% of total oxidative metabolism in the cerebrum of anesthetized rats (Patel et al., 2010; Duarte et al., 2011; Duarte and Gruetter, 2013). Furthermore, by virtue of their unique morphology and spatial distribution, astrocytes govern the transport of energy substrates from the capillary to neurons (Newington et al., 2013). The astrocytic perivascular end-feet covering the cerebral vessels (Belanger and Magistretti, 2009) structurally facilitate efficient glucose uptake into the brain via the glucose transporter 1 (GLUT1), which is expressed on both endothelial and astrocytic membrane surfaces. The astrocytic perisynaptic end-feet (Morgello et al., 1995; Haber et al., 2006) enable the astrocytes to sense neighboring synaptic activity and respond by signaling for the appropriate metabolic supply from the vascular system (Magistretti, 2011). Moreover, astrocytes are the primary cells that store glucose as glycogen, the major energy reserve in the brain (Vilchez et al., 2007). Based on these distinguishing features, astrocytes are able to closely communicate with surrounding neurons, and provide energetic substrates to fill neuronal energy needs with a precise spatiotemporal coordination. Furthermore, it is known that the call for astrocytes to satisfy the high energy requirements during neurotransmission is relayed by neurotransmitters released from neighboring activated neurons (Belanger et al., 2011).

The astrocyte-regulated reuptake of neurotransmitters plays a critical role in balancing neuronal excitatory/inhibitory tones and terminating neuronal activation (Muthukumar et al., 2014). In most brain areas, synaptic activity is mediated by glutamate, which is depleted from the synaptic cleft by uptake via the astrocytic excitatory amino acid transporter 1 and 2 (EAAT 1 and 2) or the neuronal EAAT3 (Rothstein et al., 1996; Bergles and Jahr, 1998). In astrocytes, the actions of EAATs function as a sensor to monitor the activity of surrounding glutamatergic neurons and as a switch to turn on the ANLS. Glutamate transport via EAATs is powered by the sodium gradient across the membrane. EAATs co-transport three Na^+^ ions and one H^+^ ion, but anti-transport one K^+^ ion, while taking up one glutamate molecule in a complete cycle of glutamate transport (Zerangue and Kavanaugh, 1996). In order to counter this glutamate transport-induced perturbation of ion homeostasis, the workload of Na^+^/K^+^ ATPase increases and significant amounts of ATP are consumed (Khatri and Man, 2013). The subsequent energy insufficiency triggers glycolysis to produce ATP by converting glucose into pyruvate and then to lactate by lactate dehydrogenase 5 (LDH5) in astrocytes. Lactate is then pumped out from astrocytes into the extracellular space via monocarboxylate transporters 1 and 4 (MCT1 and 4), and is taken up by neurons via monocarboxylate transporters (MCT2). After neuronal uptake, the lactate is further transported into the mitochondria via MCT1 and 2 located on the mitochondrial inner membrane and converted into pyruvate by LDH1, after which it may enter the citric acid cycle (Schousboe et al., 1997; Waagepetersen et al., 2000; Chih and Roberts, 2003; Hashimoto et al., 2008; Kane, 2014).

The oxidation of lactate to pyruvate within the neuronal mitochondria is coupled to NAD^+^ reduction. Therefore, increased oxidation of lactate within the mitochondria may enhance oxidative phosphorylation by providing additional NADH for the electron transport chain. Moreover, lactate produced in one astrocyte may diffuse to adjacent astrocytes via gap junctions (i.e., connexin 30 and 43, which link neighboring astrocytes) to build an astrocyte metabolic network (Rouach et al., 2008; Escartin and Rouach, 2013). The astrocyte metabolic network may enhance the spatial efficiency of the energy substrate shuttle between astrocytes and neurons. In summary, glutamate release from activated glutamatergic neurons instructs the surrounding astrocytes to provide lactate as energy source to fulfil high energy requirements during synaptic activity.

The ANLS hypothesis does not exclude direct glucose uptake and oxidation by neurons. However, it suggests that astrocyte-produced lactate is an important energetic substrate for neurons, especially in response to glutamatergic activity. By measuring glucose uptake and phosphorylation using glucose analog, 2-fluoro-2-deoxy-D-glucose, Patel et al. (2014) found that enhancing neuronal activity by treating rats with bicuculline, a competitive antagonist of GABAA receptors, increased the level of glucose phosphorylation in isolated brain nerve terminals. This study demonstrated that glucose-derived pyruvate may provide a major oxidative energy source for activated neurons. Their findings were later supported by Lundgaard et al. (2015) who used two-photon imaging of a near-infrared 2-deoxyglucose analog (2DG-IR) to monitor glucose transport in brain and found that 2DG-IR was taken up preferentially by neurons in awake mice, and that sensory stimulation induced a sharp increase of 2DG-IR uptake in neurons. Furthermore, hexokinase was highly enriched in neurons compared with astrocytes in both mouse and human cortices. The relative contribution of glucose and lactate as neuronal energetic substrates was also evaluated. In order to do so, Hyder et al. (2006) summarized a series of 13C NMR reports, which simultaneously measured the *in vivo* rate of total glutamate-glutamine cycling and neuronal glucose oxidation. The authors concluded that when the neuronal metabolic rate of glucose oxidation is 1.00 μmol/g/min, 0.31 glucose equivalents are derived from glucose directly taken up by neurons, while the other 0.69 glucose equivalents are provided by astrocyte-derived lactate (for detailed review, see Hyder et al., 2006).

Dissimilar to the interaction between astrocytes and glutamatergic neurons, the ANLS is not reported to be effectively activated by gamma-aminobutyric acid (GABA) transmission, the primary inhibitory system in the brain. GABA released into the synaptic cleft is removed by astrocytes via Na^+^/Cl^−^-dependent GABA transporters (Gadea and López-Colomé, 2001). In 2003, Pellerin and colleagues, who were the first to demonstrate that glutamate uptake stimulates the ANLS in mouse astrocytes, showed that GABA uptake into astrocytes did not trigger enhancement of astrocytic glycolysis and lactate release, possibly due to the insignificant metabolic cost of GABA uptake into astrocytes (Chatton et al., 2003). They found that GABA uptake in primary cortical astrocytes was achieved by co-transporting Na^+^ into the cells, but the increased intracellular Na^+^ level was not sufficient to trigger enhanced glycolysis in astrocytes. These results suggested that the Na^+^ influx induced by GABA uptake did not significantly disturb the ion homeostasis in astrocytes. Hence, it was not necessary for Na^+^/K^+^ ATPase to consume glycolysis-generated ATP to restore the Na^+^ concentration gradient (Chatton et al., 2003). In relation to learning and memory, glutamatergic transmission is dominant in the memory-related regions, such as the prefrontal cortex, amygdala and hippocampus. Therefore, it can be expected that the ANLS may play a critical role in memory processing.

## Astrocyte-Neuron Lactate Shuttle and Memory Formation

Because activity-dependent synaptic plasticity is involved in the formation of memory, it is reasonable to assume that the ANLS affects learning and memory by supporting synaptic plasticity (Newington et al., 2013; Yang et al., 2014). Indeed, it has been recently demonstrated that astrocyte-neuron lactate transport is required for long-term memory formation (Suzuki et al., 2011). As mentioned earlier, astrocytes are the principal cells that store glucose as glycogen. Astrocytic glycogen represents a major energy reserve in the brain during blood-glucose insufficiency (Brown et al., 2004), and can be broken down into lactate by glycogenolysis to fuel neuronal metabolism during neuronal activation (Brown et al., 2004). Suzuki et al. (2011) took the lead in demonstrating that lactate produced by glycogenolysis in astrocytes is required for memory processing in rodents. They found that learning led to a significant increase in extracellular lactate levels. Inhibiting glycogenolysis by intra-hippocampal injection of 1,4-dideoxy-1,4-imino-D-arabinitol abolished the learning-induced lactate increase and impaired long-term memory formation, but not the acquisition and short-term memory associated with an inhibitory avoidance task (Suzuki et al., 2011). They also showed that glycogen-derived lactate was essential for the maintenance of hippocampal long-term potentiation (LTP) elicited *in vivo*. Moreover, knockdown of astrocytic MCT1 or MCT4 by antisense oligodeoxynucleotides caused amnesia, which could be rescued by exogenous administration of L-lactate, but not glucose. Likewise, disrupting neuronal MCT2 also caused amnesia. However, the neuronal MCT2 deficiency-induced amnesia was unaffected by either L-lactate or glucose, suggesting that astrocyte-derived lactate is necessary for the formation of long-term memory. These findings were later supported by Newman et al. (2011) who demonstrated that direct delivery of lactate into the ventral hippocampus 5 min before testing improved scores on a 4-arm delayed spontaneous alternation task in rats. These results suggest that astrocyte-derived lactate is not only required for the memory formation, but participates in the memory retrieval as well.

Moreover, in order to form long-term memory, a series of signaling cascades must be activated to drive gene expression, protein synthesis, and the formation of new synaptic connections (Kandel, 2001; Alberini, 2009). The cAMP response element binding protein (CREB) is a nuclear protein that is thought to be activated by phosphorylation and regulate the transcription of proteins needed to stabilize the synaptic changes triggered during the formation of long-term memory (Silva et al., 1998; Nguyen, 2001; Lee and Silva, 2009). In 2001, Josselyn et al. (2001) provided the first direct evidence that overexpression of CREB in the mammalian brain enhances the formation of long-term memory. They compared rats given the same amount of fear conditioning training but presented in either a spaced manner (with intervening rest intervals) or massed training (with no or short intervening rest intervals) in producing LTM. Those animals given massed training showed no or weak memory formation in a fear-potentiated startle task. However, increasing CREB levels specifically in the basolateral amygdala significantly increased long-term memory after massed training. Their results suggest that CREB activity in the amygdala serves as a molecular switch for the formation of long-term memory in fear conditioning (Josselyn et al., 2001). Interestingly, the learning-dependent induction of CREB phosphorylation is completely blocked by inhibiting astrocytic glycogenolysis and rescued by lactate administration (Suzuki et al., 2011). This finding implies that lactate functions as a positive regulator of learning-dependent CREB phosphorylation. CREB phosphorylation is known to be regulated by three key pathways, i.e., the Ca^2+^- calcium calmodulin kinase kinase-calcium calmodulin kinase IV (Ca^2+^-CaMKK-CaMKIV) pathway, cyclic adenosine monophosphate-protein kinase A (cAMP-PKA) pathway, and the mitogen-activated protein kinase-ribosomal S6 kinase (MAPK-RSK) pathway (Lee and Silva, 2009). Recently, it was reported that lactate could promote neuronal expression of CREB-driven plasticity genes via ionotropic glutamate receptor-mediated Ca^2+^ influx and MAPK activity (Yang et al., 2014). Yang et al. (2014) showed that L-lactate applied to the mouse primary cortical neurons stimulated the expression of immediately early genes (IEGs), including Arc, c-Fos and Zif268. However, the effect of L-lactate on IEG expression was fully prevented in the presence of an MCT inhibitor, suggesting that L-lactate induced IEG expression after transport into neurons rather than by binding to the cell surface receptors. Moreover, L-lactate increased phosphorylation of the extracellular signal-regulated kinase 1/2 (Erk 1/2), a specific subset of the mammalian MAPK family that is implicated in N-methyl-D-aspartate receptor (NMDAR)-dependent neuronal plasticity (Giovannini, 2006; Zhou et al., 2009). Importantly, in the presence of either the NMDAR antagonists or Erk1/2 kinase inhibitors, the L-lactate-increased IEG expression was significantly diminished. This set of results clearly demonstrated that activation of the NMDAR and downstream Erk1/2 signaling is required for the observed L-lactate effects. They further demonstrated that L-lactate potentiated NMDAR-dependent inward currents and subsequently intracellular Ca^2+^ concentrations. It is known that intracellular calcium activates Erk signaling (Schmitt et al., 2004; Li et al., 2005) and IEG expression (Greenberg et al., 1992). Thus, L-lactate potentially promoted IEG expression and Erk1/2 phosphorylation by increasing NMDAR-mediated Ca^2+^ influx. As mentioned above, lactate oxidation to pyruvate in neuronal mitochondria is accompanied by NAD^+^ reduction to NADH. Consistent with this, the authors also found that exposure of neurons to L-lactate increased the intracellular NADH/NAD ratio. Furthermore, direct application of NADH to primary neuronal cultures enhanced IEG expression, Erk1/2 phosphorylation and intracellular Ca^2+^ level, similar to the effects of L-lactate. As expected, the NADH effects were abolished by treating with the NMDAR antagonists. Taken together, these results suggested that the increase of NADH coincident with lactate oxidation in neuronal mitochondria potentiated NMDA-mediated Ca^2+^ influx and enhanced IEG expression. Intriguingly, the effects of L-lactate on IEG expression was specific, in that equicaloric concentrations of D-lactate, L-pyruvate and D-glucose did not affect IEG expression levels (Yang et al., 2014). Besides participating in the regulation of IEGs, L-lactate was also reported to modulate the expression of the CREB-regulated neurotrophin, brain-derived neurotrophic factor (BDNF; Coco et al., 2013). BDNF belongs to a family of neurotrophic factors involved in regulating the survival and differentiation of developing neurons, as well as activity-dependent synaptic plasticity (Huang and Reichardt, 2001; Bramham and Messaoudi, 2005). According to the notion that activity-dependent synaptic plasticity is essential for the memory formation, a great body of evidence has suggested that BDNF plays a critical role in the processes of learning and memory (Lu et al., 2008; Kuczewski et al., 2010; Bekinschtein et al., 2014). Coco et al. (2013) demonstrated that high level of lactate (5–25 mM L-lactate) increased the expression and secretion of BDNF in both a human neuroblastoma cell line, SH-SY5Y, and primary human astrocytes. Overall, the accumulating findings establish that lactate not only functions as a supplemental energy source for neurons, but also as a signal transducing molecule to alter the neuronal redox status and induce the expression of plasticity-related genes. However, the specific role for lactate in memory formation remains unclear. In future investigations, it will be very interesting to elucidate the relative importance of and relationship between lactate-derived energy and lactate-induced gene expression in the processes of memory formation.

## Astrocyte-Neuron Lactate Shuttle and Aging-Related Memory Impairment

Cognitive decline is emerging as one of the most difficult health problems in the elderly population. Age alone increases the risk of stroke, AD, and dementia (Bishop et al., 2010). Even in the absence of overt disease symptom, increasing age is associated with decreasing cognitive function of varying severity in human beings. Fortunately, technical advances over the past two decades, such as improved gene expression analysis and functional brain imaging, have provided great insight into the basic molecular mechanisms and the large-scale cognitive networks of the aging human brain. Currently, it is well-accepted that impairments in neuroplasticity (Burke and Barnes, 2006) and brain energy metabolism (Bishop et al., 2010), two aspects in which lactate is highly involved, contribute to aging-related cognitive decline. Regarding neuroplasticity, aging is known to alter neuronal morphology, electrophysiological properties and IEG expression (Bishop et al., 2010; Hartzell et al., 2013). Compared to young subjects, reductions in dendritic arborization were observed in the prefrontal cortex of rats (Markham and Juraska, 2002) and human beings (de Brabander et al., 1998; Uylings and de Brabander, 2002), and a decrease in spine density was found in the hippocampal subiculum of monkeys (Uemura, 1985). Using electrophysiological measurements, enhanced L-type Ca^2+^ channel conductance was reported in aged hippocampal CA1 pyramidal cells (Thibault and Landfield, 1996). This augmented Ca^2+^ conductance may potentially disturb Ca^2+^ homeostasis in neurons and lead to plasticity deficits during aging (Toescu et al., 2004). Moreover, the field excitatory postsynaptic potential (fEPSP) recorded in the hippocampal CA1 (Landfield et al., 1986; Barnes et al., 1992; Deupree et al., 1993) and dentate gyrus (DG) is attenuated in the aged rats (Barnes, 1979). With peri-threshold stimulation protocols, impaired LTP inductions in the CA1 and DG (Moore et al., 1993; Rosenzweig et al., 1997; Barnes et al., 2000; Tombaugh et al., 2002) were detected in aged animals. It is interesting to note that if LTP was induced by high-frequency and high-current, the impaired LTP inductions in performant path-granule cell synapses (Barnes, 1979; Diana et al., 1994a,b) and CA3-CA1 Schaffer collateral synapses (Landfield and Lynch, 1977; Landfield et al., 1978) of aged rats became undetectable. Because expression and protein synthesis from plasticity genes are required for the maintenance of LTP, it is highly possible that aging may affect LTP via these processes (Pfeiffer and Huber, 2006; Bishop et al., 2010).

It has been reported that the phosphorylation of CREB, one of the most important plasticity gene transcription factors, is attenuated in the hippocampus but not in the prefrontal cortex of aged animals (Xu et al., 2010; Paramanik and Thakur, 2013). Thus, the aging effects on CREB function may be region-specific. Additionally, dysregulation of CREB phosphorylation after learning was also found in memory-impaired aged rats (Kudo et al., 2005; Monti et al., 2005; Countryman and Gold, 2007). This aging-related dysregulation of CREB phosphorylation in the hippocampus has been linked to an increase of L-type Ca^2+^ channel-mediated Ca^2+^ influx (Foster and Kumar, 2002) and decreased levels of CaMKII, CaMKIV and MAPK (Chung et al., 2002; Xu et al., 2010). Moreover, the number of CREB-binding protein positive cells was decreased in the hippocampus of the aged rats (Xu et al., 2010). These findings suggest the possibility that the CREB-dependent transcription is impaired in aged animals. In accordance with this hypothesis, the mRNA levels of CREB-induced IEGs were lower in memory-impaired aged rats than in healthy young animals after LTP-induction (Lanahan et al., 1997).

Aging is also known to affect brain metabolic status, largely through the development of mitochondrial dysfunction that is consistently observed in aged animals (Blalock et al., 2003; Lu et al., 2004; Zahn et al., 2007; Yankner et al., 2008). In addition to the progressive decline of mitochondrial gene expression, three major components of mitochondrial oxidative phosphorylation are dysregulated in the aged brain, i.e., electron transfer capacity, the inner membrane H^+^ gradient and H^+^-driven ATP synthesis (Ferrándiz et al., 1994; Navarro and Boveris, 2004, 2007; Boveris and Navarro, 2008). These mitochondrial impairments directly lead to an insufficient ATP supply and increased oxidative stress in brain cells (Beckman and Ames, 1998; Navarro and Boveris, 2007; Navarro et al., 2008). Moreover, dysfunctional mitochondria also affect lactate metabolism in the aged brain. In 2010, Ross et al. (2010) demonstrated that a high level of brain lactate is a hallmark of aging. They found that, in the brains of both normal aging mice and premature aging mitochondrial DNA mutated mice, dysfunctional mitochondria led to a metabolic shift from erobic respiration to glycolysis related to an increased ratio of LDH-A/LDH-B expression. This shift resulted in robustly increased brain lactate levels. Although it remains unclear that what kinds of cells generate the high level lactate and abnormally express LDH subtypes, their results implied that dysregulated LDH may perturb the conversion of lactate to pyruvate in the neuronal mitochondria and thus cause lactate accumulation in the aged brain. If the conversion process is suppressed within neuronal mitochondria, lactate loses the ability to supplement energy supply and induce the expression of plasticity genes. Future investigations may test this hypothesis and evaluate its relevance in aging-related memory impairments.

Several components of the ANLS are known to be altered in the memory-impaired aged brain. A decrease of GLUT1, which is mainly expressed in endothelium and astrocytes, was found in AD patients (Kalaria and Harik, 1989; Simpson et al., 1994). This observation is consistent with the previous ^18^F-fluorodeoxyglucose-PET studies which have demonstrated that AD patients have a symptom severity-correlated progressive reduction of cerebral glucose metabolism (Mosconi et al., 2004, 2005, 2008). Moreover, a decrease in brain LDH level and activity was observed in elderly subjects with AD (Liguri et al., 1990) or mild cognitive impairment (Reed et al., 2008). These findings suggested that the ANLS may be impaired in AD and mild cognitive impairment due to insufficient lactate source (i.e., glucose) and dysfunctional catalyzing enzymes (i.e., LDH). This opinion was later supported by Merlini et al. (2011) in AD animals. They selected the ArcAβ mice carrying mutant human amyloid precursor protein (APP) as their AD model, and found that the AD mice had decreased endothelial and astrocytic GLUT1expression accompanied with a reduced baseline brain glucose level. In primary astrocytes cultured from the ArcAβ mice, both the expression of MCT1 and lactate release upon neuronal stimulation were reduced. Moreover, it has been reported that the expression of brain astrocytic EAATs, which initiate the ANLS, are altered in both AD patients and animals. Among the varied subtypes of EAATs, the EAAT2 is concentrated in perisynaptic astrocytes which are responsible for 90% of glutamate uptake in the adult mammalian CNS. In human postmortem AD brain tissue, the expression of intact EAAT2 is decreased in the brain regions susceptible to AD pathology, but the level of a pathology-specific alternative splice variant of EAAT2 is increased (Scott et al., 2011). Reduced expression of the astrocytic EAAT1 and 2 was also detected in the hippocampus and cortex of 8-month-old APP23 mice (Schallier et al., 2011), another frequently used AD transgenic model. Interestingly, these two major astrocytic glutamate transporters were reported to be dysregulated in tangle-bearing neurons in the AD patients (Scott et al., 2002; Thai, 2002). The neurons with aberrant expression of EAAT1 were concentrated in the cortical pyramidal layer (Scott et al., 2002); however, the EAAT2-expressing neurons were identified in multiple regions, including the cortical pyramidal layer, basal nucleus of Meynert, substantia nigra, paraventricular nucleus of the hypothalamus and locus coeruleus (Thai, 2002). Although there is no direct evidence to suggest that the abnormal expression of EAATs impairs the ANLS and furthers the progression of AD pathology, this damaged glutamate reuptake system has been suggested to contribute to glutamate-mediated excitotoxicity in AD (Scott et al., 2002; Thai, 2002).

## Exercise, Astrocyte-Neuron Lactate Shuttle and Aging-Related Memory Impairment

It is widely-accepted that increased physical activity exerts multiple benefits on the brain function (Cotman et al., 2007; Lange-Asschenfeldt and Kojda, 2008; Lustig et al., 2009; Brown et al., 2013; Voss et al., 2013), including the improvement of memory (Lustig et al., 2009). For example, long-term exercise can ameliorate the aging-induced impairment of hippocampal neurogenesis which is known to be involved in memory processing (Deng et al., 2010; Yang et al., 2015). Yang et al. (2015) recently demonstrated that hippocampal neurogenesis dramatically decreased by the time mice reached nine months of age. They also found that 5-week treadmill running was able to attenuate the decline of the number of neural stem/progenitor cells (NSC) during aging, and to enhance the maturation of new-born neurons. Furthermore, it was recently reported that in APP/PS1 double transgenic AD mice, 10 weeks of treadmill exercise prevented the AD-induced memory deficits in both contextual and cued fear conditioning tasks, and increased the dendritic complexity of the hippocampal and amygdalar neurons which respectively govern these two learning tasks (Lin et al., 2015). Additionally, this long-term training also led to reduced concentrations of Aβ_40_ and Aβ_42_ in the hippocampus and amygdala of the double transgenic mice, partially via an exercise-induced increase of the expression of LRP1, a key molecule responsible for Aβ clearance (Lin et al., 2015). In humans, a longitudinal study followed 716 elderly individuals for 4 years and concluded that a higher level of physical activity correlates with reduced risk for AD (Buchman et al., 2012). Exercise is also known to activate neurons in various brain regions, especially the hippocampus (Lee et al., 2003; Clark et al., 2011). This exercise-induced hippocampal activation has been linked to the exercise-exerted benefits on hippocampal-related memory functions. Moreover, an increase of hippocampal CREB phosphorylation and plasticity gene expression accompanies hippocampal activation during exercise (Lee et al., 2003; Chen and Russo-Neustadt, 2009; Clark et al., 2011). Among the plasticity genes up-regulated by exercise, enhanced BDNF signaling plays a very important role in exercise-improved memory function (Hillman et al., 2008). In the past two decades, several studies have uncovered the mechanism by which exercise elevates BDNF expression in the brain. In addition to activity-dependent transcriptional regulation (Shieh and Ghosh, 1999), it is known that exercise is able to regulate the BDNF level by increasing circulating factors, such as insulin-like growth factor 1 (Carro et al., 2000; Nishijima et al., 2010) and irisin (Wrann et al., 2013), which can promote BDNF expression in the hippocampus. Furthermore, it is possible that exercise up-regulates brain BDNF expression through the increased production of either central or peripheral lactate. The level of lactate derived from the astrocytes in regions rich in glutamatergic neurons, such as the hippocampus, may increase due to local neuronal activation induced by exercise (Dalsgaard, 2006). Moreover, cerebral uptake of lactate is known to increase in response to the excessive systemic lactate generated form contracting muscles during exercise (Ide et al., 2000; Quistorff et al., 2008; van Hall et al., 2009; Rasmussen et al., 2011; Overgaard et al., 2012). However, the increase of brain lactate during or after exercise is region specific and may be governed by specific regulation of exercise-enhanced lactate transport. In 2014, Takimoto and Hamada (2014) demonstrated that acute exercise increases brain region-specific expression of MCTs. After exposure to a single bout of treadmill training for 2 h at a moderate intensity (20 m/min, 8% grade), compared to sedentary controls, the exercising rats had a higher levels of lactate in the cortex, hippocampus, and hypothalamus, but not the brainstem. The authors also found that the MCT1 level increased in the cortex and hippocampus, and MCT2 expression increased in the cortex, hypothalamus and hippocampus after exercise. The regional up-regulation of MCT2 after exercise was related to the exercise-induced expression of BDNF and its receptor, TrkB. Moreover, the expression of MCT4 increased only in the hypothalamus. However, none of the MCT isoforms were altered in the brainstem after exercise. This finding was consistent with the unchanged brainstem lactate level after the acute exercise. In addition to MCTs, exercise has been reported to affect the expression of EAATs. Two weeks of treadmill exercise was found to up-regulate EAAT2 and prevent reperfusion-induced excitotoxicity by removing excess glutamate from the synaptic cleft in a rat model of ischemic brain injury (Yang et al., 2012). Although an accumulating body of evidence suggests that exercise can increase the brain lactate level and the expression of ANLS components, it still remains unclear whether the increased level results from an enhanced ANLS system or increased uptake from circulation during exercise. Further, it is not known whether lactate is essential for exercise-enhanced plasticity gene expression and cognitive functions.

## Conclusion

Brain metabolic dysfunction is highly related to aging-associated dementia. Here, we have reviewed the complex mechanisms of brain energy supply and demand with an emphasis on summarizing current knowledge of the ANLS and the effects of aging and exercise on the ANLS system. A central feature of the ANLS hypothesis, that neurons consume lactate as the primary energy source during neuronal activation, remains controversial (Chih and Roberts, 2003; Lundgaard et al., 2015). However, current understanding implicates lactate as at least a supplementary metabolite, and as an important molecule involved in plasticity-related gene expression. Although brain lactate metabolism is altered by both aging and exercise, it is necessary to address whether lactate directly participates in the effects of aging and exercise on memory function. This will be important to extend our understanding of how to target lactate metabolism to prevent or reverse aging-related memory decline. Moreover, existing evidence suggests that lactate functions as an intracellular signal transducer. The surface receptor for lactate, HCA1, has been reported to be expressed in the brain (Tang et al., 2014; Mosienko et al., 2015). HCA1, previously known as GPR81, is a G_i_ protein-coupled receptor (Liu et al., 2009; Kuei et al., 2011) which inhibits adenylate cyclase and decreases cAMP level. However, the role of lactate-HCA1 signaling in the memory processes remains unclear and deserves further investigation.

## Author Contributions

S-FT: literature review and main manuscript preparation; P-CC: manuscript preparation; MJC: English editing; S-YW: manuscript preparation; Y-MK: manuscript preparation and supervision.

## Acknowledgments

This work was supported by grant (MOST 104-2320-B-006-039-MY3) from Ministry of Science and Technology, Taiwan.

## Conflict of Interest Statement

The authors declare that the research was conducted in the absence of any commercial or financial relationships that could be construed as a potential conflict of interest. The reviewer VEM and handling Editor declared a current collaboration and the handling Editor states that the process nevertheless met the standards of a fair and objective review.

## References

[B1] Alberini C. M. (2009). Transcription factors in long-term memory and synaptic plasticity. Physiol. Rev. 89, 121–145. 10.1152/physrev.00017.2008 19126756 PMC3883056

[B2] Alle H. Roth A. Geiger J. R. (2009). Energy-efficient action potentials in hippocampal mossy fibers. Science 325, 1405–1408. 10.1126/science.1174331 19745156

[B3] Attwell D. Laughlin S. B. (2001). An energy budget for signaling in the grey matter of the brain. J. Cereb. Blood Flow Metab. 21, 1133–1145. 10.1097/00004647-200110000-00001 11598490

[B4] Barnes C. A. (1979). Memory deficits associated with senescence: a neurophysiological and behavioral study in the rat. J. Comp. Physiol. Psychol. 93, 74–104. 10.1037/h0077579 221551

[B6] Barnes C. A. Rao G. Foster T. C. McNaughton B. L. (1992). Region-specific age effects on AMPA sensitivity: electrophysiological evidence for loss of synaptic contacts in hippocampal field CA1. Hippocampus 2, 457–468. 10.1002/hipo.450020413 1284976

[B5] Barnes C. A. Rao G. Houston F. P. (2000). LTP induction threshold change in old rats at the perforant path–granule cell synapse. Neurobiol. Aging 21, 613–620. 10.1016/s0197-4580(00)00163-9 11016529

[B7] Beckman K. B. Ames B. N. (1998). The free radical theory of aging matures. Physiol. Rev. 78, 547–581. 9562038 10.1152/physrev.1998.78.2.547

[B8] Bekinschtein P. Cammarota M. Medina J. H. (2014). BDNF and memory processing. Neuropharmacology 76, 677–683. 10.1016/j.neuropharm.2013.04.024 23688925

[B9] Belanger M. Allaman I. Magistretti P. J. (2011). Brain energy metabolism: focus on astrocyte-neuron metabolic cooperation. Cell Metab. 14, 724–738. 10.1016/j.cmet.2011.08.016 22152301

[B10] Belanger M. Magistretti P. J. (2009). The role of astroglia in neuroprotection. Dialogues Clin. Neurosci. 11, 281–295. 19877496 10.31887/DCNS.2009.11.3/mbelanger PMC3181926

[B11] Bergles D. E. Jahr C. E. (1998). Glial contribution to glutamate uptake at Schaffer collateral-commissural synapses in the hippocampus. J. Neurosci. 18, 7709–7716. 9742141 10.1523/JNEUROSCI.18-19-07709.1998 PMC6792997

[B12] Bishop N. A. Lu T. Yankner B. A. (2010). Neural mechanisms of ageing and cognitive decline. Nature 464, 529–535. 10.1038/nature08983 20336135 PMC2927852

[B13] Blalock E. M. Chen K. C. Sharrow K. Herman J. P. Porter N. M. Foster T. C.. (2003). Gene microarrays in hippocampal aging: statistical profiling identifies novel processes correlated with cognitive impairment. J. Neurosci. 23, 3807–3819. 12736351 10.1523/JNEUROSCI.23-09-03807.2003 PMC6742177

[B14] Bouzier-Sore A. K. Voisin P. Bouchaud V. Bezancon E. Franconi J. M. Pellerin L. (2006). Competition between glucose and lactate as oxidative energy substrates in both neurons and astrocytes: a comparative NMR study. Eur. J. Neurosci. 24, 1687–1694. 10.1111/j.1460-9568.2006.05056.x 17004932

[B15] Boveris A. Navarro A. (2008). Brain mitochondrial dysfunction in aging. IUBMB Life 60, 308–314. 10.1002/iub.46 18421773

[B16] Bramham C. R. Messaoudi E. (2005). BDNF function in adult synaptic plasticity: the synaptic consolidation hypothesis. Prog. Neurobiol. 76, 99–125. 10.1016/j.pneurobio.2005.06.003 16099088

[B17] Brown A. M. Baltan Tekkök S. Ransom B. R. (2004). Energy transfer from astrocytes to axons: the role of CNS glycogen. Neurochem. Int. 45, 529–536. 10.1016/j.neuint.2003.11.005 15186919

[B18] Brown B. M. Peiffer J. J. Martins R. N. (2013). Multiple effects of physical activity on molecular and cognitive signs of brain aging: can exercise slow neurodegeneration and delay Alzheimer’s disease? Mol. Psychiatry 18, 864–874. 10.1038/mp.2012.162 23164816

[B19] Buchman A. S. Boyle P. A. Yu L. Shah R. C. Wilson R. S. Bennett D. A. (2012). Total daily physical activity and the risk of AD and cognitive decline in older adults. Neurology 78, 1323–1329. 10.1212/WNL.0b013e3182535d35 22517108 PMC3335448

[B20] Burke S. N. Barnes C. A. (2006). Neural plasticity in the ageing brain. Nat. Rev. Neurosci. 7, 30–40. 10.1038/nrn1809 16371948

[B21] Carro E. Nuñez A. Busiguina S. Torres-Aleman I. (2000). Circulating insulin-like growth factor I mediates effects of exercise on the brain. J. Neurosci. 20, 2926–2933. 10751445 10.1523/JNEUROSCI.20-08-02926.2000 PMC6772191

[B22] Chatton J. Y. Pellerin L. Magistretti P. J. (2003). GABA uptake into astrocytes is not associated with significant metabolic cost: implications for brain imaging of inhibitory transmission. Proc. Natl. Acad. Sci. U S A 100, 12456–12461. 10.1073/pnas.2132096100 14530410 PMC218779

[B23] Chen M. J. Russo-Neustadt A. A. (2009). Running exercise-induced up-regulation of hippocampal brain-derived neurotrophic factor is CREB-dependent. Hippocampus 19, 962–972. 10.1002/hipo.20579 19294650 PMC2756465

[B24] Chih C. P. Roberts E. L. Jr. (2003). Energy substrates for neurons during neural activity: a critical review of the astrocyte-neuron lactate shuttle hypothesis. J. Cereb. Blood Flow Metab. 23, 1263–1281. 10.1097/01.wcb.0000081369.51727.6f 14600433

[B25] Chung Y. H. Kim E. J. Shin C. M. Joo K. M. Kim M. J. Woo H. W.. (2002). Age-related changes in CREB binding protein immunoreactivity in the cerebral cortex and hippocampus of rats. Brain Res. 956, 312–318. 10.1016/s0006-8993(02)03562-x 12445700

[B26] Clark P. J. Bhattacharya T. K. Miller D. S. Rhodes J. S. (2011). Induction of c-Fos, Zif268 and Arc from acute bouts of voluntary wheel running in new and pre-existing adult mouse hippocampal granule neurons. Neuroscience 184, 16–27. 10.1016/j.neuroscience.2011.03.072 21497182 PMC3100453

[B27] Coco M. Caggia S. Musumeci G. Perciavalle V. Graziano A. C. Pannuzzo G.. (2013). Sodium L-lactate differently affects brain-derived neurothrophic factor, inducible nitric oxide synthase and heat shock protein 70 kDa production in human astrocytes and SH-SY5Y cultures. J. Neurosci. Res. 91, 313–320. 10.1002/jnr.23154 23172800

[B28] Cotman C. W. Berchtold N. C. Christie L. A. (2007). Exercise builds brain health: key roles of growth factor cascades and inflammation. Trends Neurosci. 30, 464–472. 10.1016/j.tins.2007.06.011 17765329

[B29] Countryman R. A. Gold P. E. (2007). Rapid forgetting of social transmission of food preferences in aged rats: relationship to hippocampal CREB activation. Learn. Mem. 14, 350–358. 10.1101/lm.524907 17522026 PMC1876759

[B30] Dalsgaard M. K. (2006). Fuelling cerebral activity in exercising man. J. Cereb. Blood Flow Metab. 26, 731–750. 10.1038/sj.jcbfm.9600256 16395281

[B31] de Brabander J. M. Kramers R. J. Uylings H. B. (1998). Layer-specific dendritic regression of pyramidal cells with ageing in the human prefrontal cortex. Eur. J. Neurosci. 10, 1261–1269. 10.1046/j.1460-9568.1998.00137.x 9749780

[B32] Deng W. Aimone J. B. Gage F. H. (2010). New neurons and new memories: how does adult hippocampal neurogenesis affect learning and memory? Nat. Rev. Neurosci. 11, 339–350. 10.1038/nrn2822 20354534 PMC2886712

[B33] Deupree D. L. Bradley J. Turner D. A. (1993). Age-related alterations in potentiation in the CA1 region in F344 rats. Neurobiol. Aging 14, 249–258. 10.1016/0197-4580(93)90009-z 8321393

[B34] Diamond J. S. (2005). Deriving the glutamate clearance time course from transporter currents in CA1 hippocampal astrocytes: transmitter uptake gets faster during development. J. Neurosci. 25, 2906–2916. 10.1523/JNEUROSCI.5125-04.2005 15772350 PMC6725141

[B35] Diana G. Domenici M. R. Loizzo A. Scotti de Carolis A. Sagratella S. (1994a). Age and strain differences in rat place learning and hippocampal dentate gyrus frequency-potentiation. Neurosci. Lett. 171, 113–116. 10.1016/0304-3940(94)90618-1 8084469

[B36] Diana G. Scotti de Carolis A. Frank C. Domenici M. R. Sagratella S. (1994b). Selective reduction of hippocampal dentate frequency potentiation in aged rats with impaired place learning. Brain Res. Bull. 35, 107–111. 10.1016/0361-9230(94)90089-2 7953765

[B37] Duarte J. M. Gruetter R. (2013). Glutamatergic and GABAergic energy metabolism measured in the rat brain by ^13^C NMR spectroscopy at 14.1 T. J. Neurochem. 126, 579–590. 10.1111/jnc.12333 23745684

[B38] Duarte J. M. Lanz B. Gruetter R. (2011). Compartmentalized cerebral metabolism of [1,6–^13^C glucose determined by *in vivo* ^13^C NMR spectroscopy at 14.1 T. Front. Neuroenergetics 3:3. 10.3389/fnene.2011.00003 21713114 PMC3112327

[B39] Escartin C. Rouach N. (2013). Astroglial networking contributes to neurometabolic coupling. Front. Neuroenergetics 5:4. 10.3389/fnene.2013.00004 23637659 PMC3636502

[B40] Fernández-Tomé P. Brera B. Arévalo M. A. de Ceballos M. L. (2004). Beta-amyloid25–35 inhibits glutamate uptake in cultured neurons and astrocytes: modulation of uptake as a survival mechanism. Neurobiol. Dis. 15, 580–589. 10.1016/j.nbd.2003.12.006 15056466

[B41] Ferrándiz M. L. Martínez M. De Juan E. Díez A. Bustos G. Miquel J. (1994). Impairment of mitochondrial oxidative phosphorylation in the brain of aged mice. Brain Res. 644, 335–338. 10.1016/0006-8993(94)91699-3 8050045

[B42] Figley C. R. Stroman P. W. (2011). The role(s) of astrocytes and astrocyte activity in neurometabolism, neurovascular coupling and the production of functional neuroimaging signals. Eur. J. Neurosci. 33, 577–588. 10.1111/j.1460-9568.2010.07584.x 21314846

[B43] Foster T. C. Kumar A. (2002). Calcium dysregulation in the aging brain. Neuroscientist 8, 297–301. 10.1177/107385840200800404 12194497

[B44] Fox P. T. Raichle M. E. (1986). Focal physiological uncoupling of cerebral blood flow and oxidative metabolism during somatosensory stimulation in human subjects. Proc. Natl. Acad. Sci. U S A 83, 1140–1144. 10.1073/pnas.83.4.1140 3485282 PMC323027

[B45] Fox P. T. Raichle M. E. Mintun M. A. Dence C. (1988). Nonoxidative glucose consumption during focal physiologic neural activity. Science 241, 462–464. 10.1126/science.3260686 3260686

[B46] Frizzo M. E. Schwarzbold C. Porciúncula L. O. Dalcin K. B. Rosa R. B. Ribeiro C. A.. (2004). 3-hydroxyglutaric acid enhances glutamate uptake into astrocytes from cerebral cortex of young rats. Neurochem. Int. 44, 345–353. 10.1016/s0197-0186(03)00169-4 14643752

[B47] Gadea A. López-Colomé A. M. (2001). Glial transporters for glutamate, glycine and GABA: II. GABA transporters. J. Neurosci. Res. 63, 461–468. 10.1002/jnr.1040 11241581

[B48] Giovannini M. G. (2006). The role of the extracellular signal-regulated kinase pathway in memory encoding. Rev. Neurosci. 17, 619–634. 10.1515/revneuro.2006.17.6.619 17283607

[B49] Greenberg M. E. Thompson M. A. Sheng M. (1992). Calcium regulation of immediate early gene transcription. J. Physiol. Paris 86, 99–108. 10.1016/s0928-4257(05)80013-0 1343600

[B50] Haber M. Zhou L. Murai K. K. (2006). Cooperative astrocyte and dendritic spine dynamics at hippocampal excitatory synapses. J. Neurosci. 26, 8881–8891. 10.1523/JNEUROSCI.1302-06.2006 16943543 PMC6675342

[B51] Hartzell A. L. Burke S. N. Hoang L. T. Lister J. P. Rodriguez C. N. Barnes C. A. (2013). Transcription of the immediate-early gene Arc in CA1 of the hippocampus reveals activity differences along the proximodistal axis that are attenuated by advanced age. J. Neurosci. 33, 3424–3433. 10.1523/JNEUROSCI.4727-12.2013 23426670 PMC3711759

[B52] Hashimoto T. Hussien R. Cho H. S. Kaufer D. Brooks G. A. (2008). Evidence for the mitochondrial lactate oxidation complex in rat neurons: demonstration of an essential component of brain lactate shuttles. PLoS One 3:e2915. 10.1371/journal.pone.0002915 18698340 PMC2488371

[B53] Herrero-Mendez A. Almeida A. Fernández E. Maestre C. Moncada S. Bolaños J. P. (2009). The bioenergetic and antioxidant status of neurons is controlled by continuous degradation of a key glycolytic enzyme by APC/C-Cdh1. Nat. Cell Biol. 11, 747–752. 10.1038/ncb1881 19448625

[B54] Hillman C. H. Erickson K. I. Kramer A. F. (2008). Be smart, exercise your heart: exercise effects on brain and cognition. Nat. Rev. Neurosci. 9, 58–65. 10.1038/nrn2298 18094706

[B55] Howarth C. Gleeson P. Attwell D. (2012). Updated energy budgets for neural computation in the neocortex and cerebellum. J. Cereb. Blood Flow Metab. 32, 1222–1232. 10.1038/jcbfm.2012.35 22434069 PMC3390818

[B56] Huang E. J. Reichardt L. F. (2001). Neurotrophins: roles in neuronal development and function. Annu. Rev. Neurosci. 24, 677–736. 10.1146/annurev.neuro.24.1.677 11520916 PMC2758233

[B57] Hyder F. Patel A. B. Gjedde A. Rothman D. L. Behar K. L. Shulman R. G. (2006). Neuronal-glial glucose oxidation and glutamatergic-GABAergic function. J. Cereb. Blood Flow Metab. 26, 865–877. 10.1038/sj.jcbfm.9600263 16407855

[B58] Ide K. Schmalbruch I. K. Quistorff B. Horn A. Secher N. H. (2000). Lactate, glucose and O2 uptake in human brain during recovery from maximal exercise. J. Physiol. 522, 159–164. 10.1111/j.1469-7793.2000.t01-2-00159.xm 10618160 PMC2269743

[B59] Itoh Y. Esaki T. Shimoji K. Cook M. Law M. J. Kaufman E.. (2003). Dichloroacetate effects on glucose and lactate oxidation by neurons and astroglia *in vitro* and on glucose utilization by brain *in vivo*. Proc. Natl. Acad. Sci. U S A 100, 4879–4884. 10.1073/pnas.0831078100 12668764 PMC153649

[B60] Jolivet R. Magistretti P. J. Weber B. (2009). Deciphering neuron-glia compartmentalization in cortical energy metabolism. Front. Neuroenergetics 1:4. 10.3389/neuro.14.004.2009 19636395 PMC2715922

[B61] Josselyn S. A. Shi C. Carlezon W. A. Jr. Neve R. L. Nestler E. J. Davis M. (2001). Long-term memory is facilitated by cAMP response element-binding protein overexpression in the amygdala. J. Neurosci. 21, 2404–2412. 11264314 10.1523/JNEUROSCI.21-07-02404.2001 PMC6762400

[B62] Kalaria R. N. Harik S. I. (1989). Reduced glucose transporter at the blood-brain barrier and in cerebral cortex in Alzheimer’s disease. J. Neurochem. 53, 1083–1088. 10.1111/j.1471-4159.1989.tb07399.x 2769254

[B63] Kandel E. R. (2001). The molecular biology of memory storage: a dialogue between genes and synapses. Science 294, 1030–1038. 10.1126/science.1067020 11691980

[B64] Kane D. A. (2014). Lactate oxidation at the mitochondria: a lactate-malate-aspartate shuttle at work. Front. Neurosci. 8:366. 10.3389/fnins.2014.00366 25505376 PMC4243568

[B65] Khatri N. Man H. Y. (2013). Synaptic activity and bioenergy homeostasis: implications in brain trauma and neurodegenerative diseases. Front. Neurol. 4:199. 10.3389/fneur.2013.00199 24376435 PMC3858785

[B66] Kuczewski N. Porcher C. Gaiarsa J. L. (2010). Activity-dependent dendritic secretion of brain-derived neurotrophic factor modulates synaptic plasticity. Eur. J. Neurosci. 32, 1239–1244. 10.1111/j.1460-9568.2010.07378.x 20880359

[B67] Kudo K. Wati H. Qiao C. Arita J. Kanba S. (2005). Age-related disturbance of memory and CREB phosphorylation in CA1 area of hippocampus of rats. Brain Res. 1054, 30–37. 10.1016/j.brainres.2005.06.045 16054117

[B68] Kuei C. Yu J. Zhu J. Wu J. Zhang L. Shih A.. (2011). Study of GPR81, the lactate receptor, from distant species identifies residues and motifs critical for GPR81 functions. Mol. Pharmacol. 80, 848–858. 10.1124/mol.111.074500 21862690

[B69] Lanahan A. Lyford G. Stevenson G. S. Worley P. F. Barnes C. A. (1997). Selective alteration of long-term potentiation-induced transcriptional response in hippocampus of aged, memory-impaired rats. J. Neurosci. 17, 2876–2885. 9092609 10.1523/JNEUROSCI.17-08-02876.1997 PMC6573119

[B70] Landfield P. W. Lynch G. (1977). Impaired monosynaptic potentiation in *in vitro* hippocampal slices from aged, memory-deficient rats. J Gerontol 32, 523–533. 10.1093/geronj/32.5.523 196001

[B71] Landfield P. W. McGaugh J. L. Lynch G. (1978). Impaired synaptic potentiation processes in the hippocampus of aged, memory-deficient rats. Brain Res. 150, 85–101. 10.1016/0006-8993(78)90655-8 208716

[B72] Landfield P. W. Pitler T. A. Applegate M. D. (1986). The effects of high Mg2+-to-Ca2+ ratios on frequency potentiation in hippocampal slices of young and aged rats. J. Neurophysiol. 56, 797–811. 3783221 10.1152/jn.1986.56.3.797

[B73] Lange-Asschenfeldt C. Kojda G. (2008). Alzheimer’s disease, cerebrovascular dysfunction and the benefits of exercise: from vessels to neurons. Exp. Gerontol. 43, 499–504. 10.1016/j.exger.2008.04.002 18474414

[B74] Lee T. H. Jang M. H. Shin M. C. Lim B. V. Kim Y. P. Kim H.. (2003). Dependence of rat hippocampal c-Fos expression on intensity and duration of exercise. Life Sci. 72, 1421–1436. 10.1016/s0024-3205(02)02406-2 12527039

[B75] Lee Y. S. Silva A. J. (2009). The molecular and cellular biology of enhanced cognition. Nat. Rev. Neurosci. 10, 126–140. 10.1038/nrn2572 19153576 PMC2664745

[B76] Li D. W. Liu J. P. Mao Y. W. Xiang H. Wang J. Ma W. Y.. (2005). Calcium-activated RAF/MEK/ERK signaling pathway mediates p53-dependent apoptosis and is abrogated by alpha B-crystallin through inhibition of RAS activation. Mol. Biol. Cell 16, 4437–4453. 10.1091/mbc.e05-01-0010 16000378 PMC1196350

[B77] Liguri G. Taddei N. Nassi P. Latorraca S. Nediani C. Sorbi S. (1990). Changes in Na^+^,K^+^-ATPase, Ca^2+^-ATPase and some soluble enzymes related to energy metabolism in brains of patients with Alzheimer’s disease. Neurosci. Lett. 112, 338–342. 10.1016/0304-3940(90)90227-Z 2163043

[B78] Lin T. W. Shih Y. H. Chen S. J. Lien C. H. Chang C. Y. Huang T. Y.. (2015). Running exercise delays neurodegeneration in amygdala and hippocampus of Alzheimer’s disease (APP/PS1) transgenic mice. Neurobiol. Learn. Mem. 118, 189–197. 10.1016/j.nlm.2014.12.005 25543023

[B79] Liu C. Wu J. Zhu J. Kuei C. Yu J. Shelton J.. (2009). Lactate inhibits lipolysis in fat cells through activation of an orphan G-protein-coupled receptor, GPR81. J. Biol. Chem. 284, 2811–2822. 10.1074/jbc.M806409200 19047060

[B81] Lu Y. Christian K. Lu B. (2008). BDNF: a key regulator for protein synthesis-dependent LTP and long-term memory? Neurobiol. Learn. Mem. 89, 312–323. 10.1016/j.nlm.2007.08.018 17942328 PMC2387254

[B80] Lu T. Pan Y. Kao S. Y. Li C. Kohane I. Chan J.. (2004). Gene regulation and DNA damage in the ageing human brain. Nature 429, 883–891. 10.1038/nature02661 15190254

[B82] Lundgaard I. Li B. Xie L. Kang H. Sanggaard S. Haswell J. D.. (2015). Direct neuronal glucose uptake Heralds activity-dependent increases in cerebral metabolism. Nat. Commun. 6:6807. 10.1038/ncomms7807 25904018 PMC4410436

[B83] Lustig C. Shah P. Seidler R. Reuter-Lorenz P. A. (2009). Aging, training and the brain: a review and future directions. Neuropsychol. Rev. 19, 504–522. 10.1007/s11065-009-9119-9 19876740 PMC3005345

[B84] Magistretti P. J. (2011). Neuron-glia metabolic coupling and plasticity. Exp. Physiol. 96, 407–410. 10.1113/expphysiol.2010.053157 21123364

[B85] Markham J. A. Juraska J. M. (2002). Aging and sex influence the anatomy of the rat anterior cingulate cortex. Neurobiol. Aging 23, 579–588. 10.1016/s0197-4580(02)00004-0 12009507

[B86] Martin S. J. Grimwood P. D. Morris R. G. (2000). Synaptic plasticity and memory: an evaluation of the hypothesis. Annu. Rev. Neurosci. 23, 649–711. 10.1146/annurev.neuro.23.1.649 10845078

[B87] Merlini M. Meyer E. P. Ulmann-Schuler A. Nitsch R. M. (2011). Vascular beta-amyloid and early astrocyte alterations impair cerebrovascular function and cerebral metabolism in transgenic arcAbeta mice. Acta Neuropathol. 122, 293–311. 10.1007/s00401-011-0834-y 21688176 PMC3168476

[B88] Monti B. Berteotti C. Contestabile A. (2005). Dysregulation of memory-related proteins in the hippocampus of aged rats and their relation with cognitive impairment. Hippocampus 15, 1041–1049. 10.1002/hipo.20099 16086428

[B89] Moore C. I. Browning M. D. Rose G. M. (1993). Hippocampal plasticity induced by primed burst, but not long-term potentiation, stimulation is impaired in area CA1 of aged Fischer 344 rats. Hippocampus 3, 57–66. 10.1002/hipo.450030106 8364683

[B90] Morgello S. Uson R. R. Schwartz E. J. Haber R. S. (1995). The human blood-brain barrier glucose transporter (GLUT1) is a glucose transporter of gray matter astrocytes. Glia 14, 43–54. 10.1002/glia.440140107 7615345

[B91] Mosconi L. Perani D. Sorbi S. Herholz K. Nacmias B. Holthoff V.. (2004). MCI conversion to dementia and the APOE genotype: a prediction study with FDG-PET. Neurology 63, 2332–2340. 10.1212/01.wnl.0000147469.18313.3b 15623696

[B92] Mosconi L. Pupi A. De Leon M. J. (2008). Brain glucose hypometabolism and oxidative stress in preclinical Alzheimer’s disease. Ann. N Y Acad. Sci. 1147, 180–195. 10.1196/annals.1427.007 19076441 PMC2661241

[B93] Mosconi L. Tsui W. H. De Santi S. Li J. Rusinek H. Convit A.. (2005). Reduced hippocampal metabolism in MCI and AD: automated FDG-PET image analysis. Neurology 64, 1860–1867. 10.1212/01.wnl.0000163856.13524.08 15955934

[B94] Mosienko V. Teschemacher A. G. Kasparov S. (2015). Is L-lactate a novel signaling molecule in the brain? J. Cereb. Blood Flow Metab. 35, 1069–1075. 10.1038/jcbfm.2015.77 25920953 PMC4640281

[B95] Muthukumar A. K. Stork T. Freeman M. R. (2014). Activity-dependent regulation of astrocyte GAT levels during synaptogenesis. Nat. Neurosci. 17, 1340–1350. 10.1038/nn.3791 25151265 PMC4176984

[B96] Navarro A. Boveris A. (2004). Rat brain and liver mitochondria develop oxidative stress and lose enzymatic activities on aging. Am. J. Physiol. Regul. Integr. Comp. Physiol. 287, R1244–R1249. 10.1152/ajpregu.00226.2004 15271654

[B97] Navarro A. Boveris A. (2007). The mitochondrial energy transduction system and the aging process. Am. J. Physiol. Cell Physiol. 292, C670–C686. 10.1152/ajpcell.00213.2006 17020935

[B98] Navarro A. López-Cepero J. M. Bández M. J. Sánchez-Pino M. J. Gómez C. Cadenas E.. (2008). Hippocampal mitochondrial dysfunction in rat aging. Am. J. Physiol. Regul. Integr. Comp. Physiol. 294, R501–R509. 10.1152/ajpregu.00492.2007 18077512

[B99] Newington J. T. Harris R. A. Cumming R. C. (2013). Reevaluating metabolism in Alzheimer’s disease from the perspective of the astrocyte-neuron lactate shuttle model. J. Neurodegener. Dis. 2013:234572. 10.1155/2013/234572 26316984 PMC4437330

[B100] Newman L. A. Korol D. L. Gold P. E. (2011). Lactate produced by glycogenolysis in astrocytes regulates memory processing. PLoS One 6:e28427. 10.1371/journal.pone.0028427 22180782 PMC3236748

[B101] Nguyen P. V. (2001). CREB and the enhancement of long-term memory. Trends Neurosci. 24:314. 10.1016/s0166-2236(00)01872-5 11356494

[B102] Nishijima T. Piriz J. Duflot S. Fernandez A. M. Gaitan G. Gomez-Pinedo U.. (2010). Neuronal activity drives localized blood-brain-barrier transport of serum insulin-like growth factor-I into the CNS. Neuron 67, 834–846. 10.1016/j.neuron.2010.08.007 20826314

[B103] Overgaard M. Rasmussen P. Bohm A. M. Seifert T. Brassard P. Zaar M.. (2012). Hypoxia and exercise provoke both lactate release and lactate oxidation by the human brain. FASEB J. 26, 3012–3020. 10.1096/fj.11-191999 22441982

[B104] Paramanik V. Thakur M. K. (2013). Role of CREB signaling in aging brain. Arch. Ital. Biol. 151, 33–42. 10.4449/aib.v151i1.1461 23807618

[B105] Patel A. B. de Graaf R. A. Rothman D. L. Behar K. L. Mason G. F. (2010). Evaluation of cerebral acetate transport and metabolic rates in the rat brain *in vivo* using 1H-[13C]-NMR. J. Cereb. Blood Flow Metab. 30, 1200–1213. 10.1038/jcbfm.2010.2 20125180 PMC2879471

[B106] Patel A. B. Lai J. C. Chowdhury G. M. Hyder F. Rothman D. L. Shulman R. G.. (2014). Direct evidence for activity-dependent glucose phosphorylation in neurons with implications for the astrocyte-to-neuron lactate shuttle. Proc. Natl. Acad. Sci. U S A 111, 5385–5390. 10.1073/pnas.1403576111 24706914 PMC3986127

[B107] Pellerin L. Magistretti P. J. (1994). Glutamate uptake into astrocytes stimulates aerobic glycolysis: a mechanism coupling neuronal activity to glucose utilization. Proc. Natl. Acad. Sci. U S A 91, 10625–10629. 10.1073/pnas.91.22.10625 7938003 PMC45074

[B108] Pellerin L. Magistretti P. J. (1997). Glutamate uptake stimulates Na^+^,K^+^-ATPase activity in astrocytes via activation of a distinct subunit highly sensitive to ouabain. J. Neurochem. 69, 2132–2137. 10.1046/j.1471-4159.1997.69052132.x 9349559

[B109] Petzold G. C. Murthy V. N. (2011). Role of astrocytes in neurovascular coupling. Neuron 71, 782–797. 10.1016/j.neuron.2011.08.009 21903073

[B110] Pfeiffer B. E. Huber K. M. (2006). Current advances in local protein synthesis and synaptic plasticity. J. Neurosci. 26, 7147–7150. 10.1523/jneurosci.1797-06.2006 16822970 PMC6673933

[B111] Quistorff B. Secher N. H. Van Lieshout J. J. (2008). Lactate fuels the human brain during exercise. FASEB J. 22, 3443–3449. 10.1096/fj.08-106104 18653766

[B112] Rasmussen P. Wyss M. T. Lundby C. (2011). Cerebral glucose and lactate consumption during cerebral activation by physical activity in humans. FASEB J. 25, 2865–2873. 10.1096/fj.11-183822 21602451

[B113] Reed T. Perluigi M. Sultana R. Pierce W. M. Klein J. B. Turner D. M.. (2008). Redox proteomic identification of 4-hydroxy-2-nonenal-modified brain proteins in amnestic mild cognitive impairment: insight into the role of lipid peroxidation in the progression and pathogenesis of Alzheimer’s disease. Neurobiol. Dis. 30, 107–120. 10.1016/j.nbd.2007.12.007 18325775

[B114] Rosenzweig E. S. Rao G. McNaughton B. L. Barnes C. A. (1997). Role of temporal summation in age-related long-term potentiation-induction deficits. Hippocampus 7, 549–558. 10.1002/(sici)1098-1063(1997)7:5<549::aid-hipo10>3.0.co;2-0 9347351

[B115] Ross J. M. Öberg J. Brené S. Coppotelli G. Terzioglu M. Pernold K.. (2010). High brain lactate is a hallmark of aging and caused by a shift in the lactate dehydrogenase A/B ratio. Proc. Natl. Acad. Sci. U S A 107, 20087–20092. 10.1073/pnas.1008189107 21041631 PMC2993405

[B116] Rothstein J. D. Dykes-Hoberg M. Pardo C. A. Bristol L. A. Jin L. Kuncl R. W.. (1996). Knockout of glutamate transporters reveals a major role for astroglial transport in excitotoxicity and clearance of glutamate. Neuron 16, 675–686. 10.1016/s0896-6273(00)80086-0 8785064

[B117] Rouach N. Koulakoff A. Abudara V. Willecke K. Giaume C. (2008). Astroglial metabolic networks sustain hippocampal synaptic transmission. Science 322, 1551–1555. 10.1126/science.1164022 19056987

[B118] Schallier A. Smolders I. Van Dam D. Loyens E. De Deyn P. P. Michotte A.. (2011). Region- and age-specific changes in glutamate transport in the AbetaPP23 mouse model for Alzheimer’s disease. J. Alzheimers Dis. 24, 287–300. 10.3233/JAD-2011-101005 21297271

[B119] Schmitt J. M. Wayman G. A. Nozaki N. Soderling T. R. (2004). Calcium activation of ERK mediated by calmodulin kinase I. J. Biol. Chem. 279, 24064–24072. 10.1074/jbc.m401501200 15150258

[B120] Schousboe A. Westergaard N. Waagepetersen H. S. Larsson O. M. Bakken I. J. Sonnewald U. (1997). Trafficking between glia and neurons of TCA cycle intermediates and related metabolites. Glia 21, 99–105. 10.1002/(sici)1098-1136(199709)21:1<99::aid-glia11>3.0.co;2-w 9298852

[B121] Scott H. A. Gebhardt F. M. Mitrovic A. D. Vandenberg R. J. Dodd P. R. (2011). Glutamate transporter variants reduce glutamate uptake in Alzheimer’s disease. Neurobiol. Aging 32, 553.e1–553.e11. 10.1016/j.neurobiolaging.2010.03.008 20416976

[B122] Scott H. L. Pow D. V. Tannenberg A. E. Dodd P. R. (2002). Aberrant expression of the glutamate transporter excitatory amino acid transporter 1 (EAAT1) in Alzheimer’s disease. J. Neurosci. 22:RC206. 11826152 10.1523/JNEUROSCI.22-03-j0004.2002 PMC6758536

[B123] Shieh P. B. Ghosh A. (1999). Molecular mechanisms underlying activity-dependent regulation of BDNF expression. J. Neurobiol. 41, 127–134. 10.1002/(sici)1097-4695(199910)41:1<127::aid-neu16>3.0.co;2-j 10504200

[B124] Silva A. J. Kogan J. H. Frankland P. W. Kida S. (1998). CREB and memory. Annu. Rev. Neurosci. 21, 127–148. 10.1146/annurev.neuro.21.1.127 9530494

[B125] Simpson I. A. Chundu K. R. Davies-Hill T. Honer W. G. Davies P. (1994). Decreased concentrations of GLUT1 and GLUT3 glucose transporters in the brains of patients with Alzheimer’s disease. Ann. Neurol. 35, 546–551. 10.1002/ana.410350507 8179300

[B126] Suzuki A. Stern S. A. Bozdagi O. Huntley G. W. Walker R. H. Magistretti P. J.. (2011). Astrocyte-neuron lactate transport is required for long-term memory formation. Cell 144, 810–823. 10.1016/j.cell.2011.02.018 21376239 PMC3073831

[B127] Takimoto M. Hamada T. (2014). Acute exercise increases brain region-specific expression of MCT1, MCT2, MCT4, GLUT1 and COX IV proteins. J. Appl. Physiol. (1985) 116, 1238–1250. 10.1152/japplphysiol.01288.2013 24610532

[B129] Tang F. Lane S. Korsak A. Paton J. F. Gourine A. V. Kasparov S.. (2014). Lactate-mediated glia-neuronal signalling in the mammalian brain. Nat. Commun. 5:3284. 10.1038/ncomms4284 24518663 PMC3926012

[B130] Thai D. R. (2002). Excitatory amino acid transporter EAAT-2 in tangle-bearing neurons in Alzheimer’s disease. Brain Pathol. 12, 405–411. 10.1111/j.1750-3639.2002.tb00457.x 12408226 PMC8095978

[B131] Thibault O. Landfield P. W. (1996). Increase in single L-type calcium channels in hippocampal neurons during aging. Science 272, 1017–1020. 10.1126/science.272.5264.1017 8638124

[B132] Toescu E. C. Verkhratsky A. Landfield P. W. (2004). Ca2+ regulation and gene expression in normal brain aging. Trends Neurosci. 27, 614–620. 10.1016/j.tins.2004.07.010 15374673

[B133] Tombaugh G. C. Rowe W. B. Chow A. R. Michael T. H. Rose G. M. (2002). Theta-frequency synaptic potentiation in CA1 *in vitro* distinguishes cognitively impaired from unimpaired aged Fischer 344 rats. J. Neurosci. 22, 9932–9940. 12427850 10.1523/JNEUROSCI.22-22-09932.2002 PMC6757825

[B134] Tramontina F. Tramontina A. C. Souza D. F. Leite M. C. Gottfried C. Souza D. O.. (2006). Glutamate uptake is stimulated by extracellular S100B in hippocampal astrocytes. Cell. Mol. Neurobiol. 26, 81–86. 10.1007/s10571-006-9099-8 16633903 PMC11521381

[B135] Uemura E. (1985). Age-related changes in the subiculum of Macaca mulatta: dendritic branching pattern. Exp. Neurol. 87, 412–427. 10.1016/0014-4886(85)90172-4 3972045

[B136] Uylings H. B. de Brabander J. M. (2002). Neuronal changes in normal human aging and Alzheimer’s disease. Brain Cogn. 49, 268–276. 10.1006/brcg.2001.1500 12139954

[B137] van Hall G. Strømstad M. Rasmussen P. Jans O. Zaar M. Gam C.. (2009). Blood lactate is an important energy source for the human brain. J. Cereb. Blood Flow Metab. 29, 1121–1129. 10.1038/jcbfm.2009.35 19337275

[B138] Vilchez D. Ros S. Cifuentes D. Pujadas L. Vallès J. García-Fojeda B.. (2007). Mechanism suppressing glycogen synthesis in neurons and its demise in progressive myoclonus epilepsy. Nat. Neurosci. 10, 1407–1413. 10.1038/nn1998 17952067

[B139] Voss M. W. Vivar C. Kramer A. F. van Praag H. (2013). Bridging animal and human models of exercise-induced brain plasticity. Trends Cogn. Sci. 17, 525–544. 10.1016/j.tics.2013.08.001 24029446 PMC4565723

[B140] Waagepetersen H. S. Sonnewald U. Larsson O. M. Schousboe A. (2000). A possible role of alanine for ammonia transfer between astrocytes and glutamatergic neurons. J. Neurochem. 75, 471–479. 10.1046/j.1471-4159.2000.0750471.x 10899921

[B141] Wrann C. D. White J. P. Salogiannnis J. Laznik-Bogoslavski D. Wu J. Ma D.. (2013). Exercise induces hippocampal BDNF through a PGC-1alpha/FNDC5 pathway. Cell Metab. 18, 649–659. 10.1016/j.cmet.2013.09.008 24120943 PMC3980968

[B142] Xu J. Rong S. Xie B. Sun Z. Deng Q. Wu H.. (2010). Memory impairment in cognitively impaired aged rats associated with decreased hippocampal CREB phosphorylation: reversal by procyanidins extracted from the lotus seedpod. J. Gerontol. A Biol. Sci. Med. Sci. 65, 933–940. 10.1093/gerona/glq094 20530246

[B145] Yang X. He Z. Zhang Q. Wu Y. Hu Y. Wang X.. (2012). Pre-ischemic treadmill training for prevention of ischemic brain injury via regulation of glutamate and its transporter GLT-1. Int. J. Mol. Sci. 13, 9447–9459. 10.3390/ijms13089447 22949807 PMC3431805

[B144] Yang T. T. Lo C. P. Tsai P. S. Wu S. Y. Wang T. F. Chen Y. W.. (2015). Aging and exercise affect hippocampal neurogenesis via different mechanisms. PLoS One 10:e0132152. 10.1371/journal.pone.0132152 26147302 PMC4493040

[B143] Yang J. Ruchti E. Petit J. M. Jourdain P. Grenningloh G. Allaman I.. (2014). Lactate promotes plasticity gene expression by potentiating NMDA signaling in neurons. Proc. Natl. Acad. Sci. U S A 111, 12228–12233. 10.1073/pnas.1322912111 25071212 PMC4143009

[B146] Yankner B. A. Lu T. Loerch P. (2008). The aging brain. Annu. Rev. Pathol. 3, 41–66. 10.1146/annurev.pathmechdis.2.010506.092044 18039130

[B147] Zahn J. M. Poosala S. Owen A. B. Ingram D. K. Lustig A. Carter A.. (2007). AGEMAP: a gene expression database for aging in mice. PLoS Genet. 3:e201. 10.1371/journal.pgen.0030201.eor 18081424 PMC2098796

[B148] Zeng X. N. Sun X. L. Gao L. Fan Y. Ding J. H. Hu G. (2007). Aquaporin-4 deficiency down-regulates glutamate uptake and GLT-1 expression in astrocytes. Mol. Cell. Neurosci. 34, 34–39. 10.1016/j.mcn.2006.09.008 17074507

[B149] Zerangue N. Kavanaugh M. P. (1996). Flux coupling in a neuronal glutamate transporter. Nature 383, 634–637. 10.1038/383634a0 8857541

[B150] Zhou X. Moon C. Zheng F. Luo Y. Soellner D. Nunez J. L.. (2009). N-methyl-D-aspartate-stimulated ERK1/2 signaling and the transcriptional up-regulation of plasticity-related genes are developmentally regulated following *in vitro* neuronal maturation. J. Neurosci. Res. 87, 2632–2644. 10.1002/jnr.22103 19396876 PMC2857719

[B151] Zlokovic B. V. (2011). Neurovascular pathways to neurodegeneration in Alzheimer’s disease and other disorders. Nat. Rev. Neurosci. 12, 723–738. 10.1038/nrn3114 22048062 PMC4036520

